# Spirituality and Religion in Older Adulthood: A Scoping Review of Research Variables and Measures

**DOI:** 10.1093/geroni/igaf122.1050

**Published:** 2025-12-31

**Authors:** Shaowei Wan, Stephen Fogle

**Affiliations:** Palo Alto VA Hospital, Palo Alto, California, United States; At-Large, Omaha, Nebraska, United States

## Abstract

Publication metrics in epidemiological research reveal surging interest in religion, spirituality, and aging. It is important that operationalization of these dynamic and multi-faceted phenomena reflected their expressions among older adult populations. To this end, this paper offers a scoping review to understand religion and spirituality variables as found in population health data commonly used for older adult research. This study uses the Arksey and O’Malley scoping review framework to systematically search for articles in MEDLINE, PubMed, Cochrane Library, CINAHL, and PsycINFO (including Psych Test) during March of 2025. Articles published from 2015 to the present will be included for the review. Results will be presented according to the following thematic categories: religious and spiritual traditions, practices, experiences, and popular theologies. The insights offered by this scoping review provide an updated toolkit for epidemiological aging-related research in religion and spirituality. Specifically, the results illuminate the depth and diversity of religion and spirituality in the lived experiences of older adults. Finally, the paper delivers cogent recommendations for implementation of religion and spirituality research across pluralistic populations.

